# Raw milk and the rejection of food safety

**DOI:** 10.1002/jsfa.71049

**Published:** 2026-09-03

**Authors:** Yaohua Feng

**Affiliations:** ^1^ Department of Food Science, College of Agriculture Purdue University West Lafayette Indiana USA

**Keywords:** consumer perception, dairy, food safety, human factors, trust

## Abstract

Raw milk remains popular among consumers despite recent outbreaks of foodborne illnesses and public health warnings. Raw milk consumption is not simply consumer rejection of food safety, but a reflection of how consumers interpret food safety. We examine how trust, beliefs, and values affect consumers' decisions about raw milk consumption and how to build trust with consumers through greater transparency and engagement. © 2026 The Author(s). *Journal of the Science of Food and Agriculture* published by John Wiley & Sons Ltd on behalf of Society of Chemical Industry.

Despite decades of scientific evidence demonstrating the risks associated with consuming raw milk, there is a consistent demand for raw milk in the USA. Several states in the USA have reduced their regulations on the sale of raw milk to consumers, from 30 states permitting some form of raw milk sales in 2011 to 38 states by 2019 despite the risks.^1^ A recent multistate outbreak of *Salmonella* Typhimurium that was linked to raw milk made 171 people sick, with an 18% hospitalization rate among those under the age of 20.^2^ This contradiction highlights a human factors question: if the microbiological risks are well established, why do consumers continue to choose raw milk and reject food safety?

Consumers' food‐related decisions are shaped not only by scientific evidence but also by human factors formed from their personal values. When making decisions regarding consumption of raw milk, consumers tend to evaluate a different set of benefits and risks other than those presented by food safety professionals. While food safety professionals primarily focus on reducing microbiological and chemical hazards, consumers often evaluate food through a broader lens that includes naturalness, nutritional quality, price, and trustworthiness.^3^ Raw milk consumption represents more than a food safety issue; it serves as a case study for understanding how consumer perceptions and values can diverge from scientific risk assessments.

For many consumers, food safety is considered a baseline expectation rather than an attribute, and information about ingredients, processing methods, and production practices tends to influence their purchasing decisions.^4^ When assessing the risk of food technologies, consumers are concerned about potential health risks but care more about the qualities that they feel processing may remove. For example, they may prioritize the preservation of nutrients, sensory attributes, and perceived product integrity. To consumers, the question is less about ‘Will this food make me sick?’ but more about ‘What is lost when this food is processed?’

When looking more closely at what shapes consumers' food‐based decisions, aside from differing definitions of food safety, consumers also may rely on a series of cognitive heuristics when evaluating a particular food choice. These mental shortcuts help simplify complex food choices but may lead to perceptions that differ from scientific risk assessments.^1^ In the context of raw milk, heuristics related to naturalness, local food systems, and price shape consumers' decision‐making.

The Naturalness Heuristic dictates that consumers perceive raw milk not as a higher‐risk product, but as a more authentic or natural one. The popularity of raw milk coincides with broader cultural movements favoring natural, minimally processed, and farm‐to‐table foods.^5^ Consumers often associate these products with higher nutritional quality, greater authenticity, and improved safety.^6^

The Local Foods Heuristic aligns with the trend that raw milk is frequently sold through local farms, specialty retailers, and artisan supply chains. For many consumers, the motivation to purchase raw milk extends beyond the product itself to a commitment to local food systems and community values.^1^ Trust in local producers may serve as a shortcut for evaluating food quality and safety, particularly when consumers have direct interactions with farmers, or perceive greater transparency within local food networks.

Finally, the Price‐Quality Heuristic extends the favoring of local farms, specialty retailers, and artisan supply chains to their higher production costs, resulting in higher prices. This price premium often functions as a heuristic for quality, in which consumers mistakenly equate the elevated cost of artisanal or unpasteurized products with superior health outcomes.^7^ Consumers often use prices as a signal of food quality.^8^

It is clear from this evidence that the continuing consumption of raw milk is largely motivated by consumers' personal beliefs and values that shape how they interpret food. An additional human factor that cannot be avoided when analyzing the raw milk trend is the loss of trust in modern food systems. This loss of trust causes consumers to view pasteurization as a corrective measure for industry failures. As food safety professionals, we know that pasteurization is a protective layer that reduces risk, even when production follows good manufacturing practices.

This loss of trust is a human factor that has had a significant impact on consumer decision‐making in my previous studies. Foodborne outbreaks and recalls are reported frequently. Unfortunately, when consumers hear about these events, the information often reaches them not as an objective fact, but through media coverage or even documentary films, which can carry strong emotion and shape a more subjective agenda.^9^ Gozzi *et al*. (under review) found that young adults (18–24 years) who viewed the food safety documentary ‘Poisoned: The Dirty Truth About Your Food’ (directed by Stephanie Soechtig) reported reduced trust in the food industry and regulatory stakeholders. These regulatory failures can erode trust not only in the specific commodities featured, but also in the food system as a whole.

The 2022 powdered infant formula recall provides an example of why consumers may have distrust in food systems. To many consumers, powdered infant formula is believed to be manufactured for one of the most vulnerable population groups and sold through mainstream retailers. When the powdered infant formula *Cronobacter* outbreaks led to widespread product shortages and empty store shelves,^10^ many parents were confronted with a troubling reality: even processed foods designed for vulnerable infants can become contaminated with harmful pathogens. For some consumers, this type of failure may reinforce the belief that modern food systems are too distant and complex to be trusted. If a product made for babies can fail, consumers may begin to question what food processing and regulations guarantee. This loss of trust can be further amplified by health and wellness influencers, who often present personal experiences or ‘their truth’ about the food industry as if they are equally valid as scientific evidence. These messages may be especially persuasive when they appear personal, emotional, and relatable, even when they are not supported by food safety research. This leads to a simple question for some consumers: if modern processing can fail, why not return to the traditional and more natural way of serving milk, which is raw milk?

From an industry perspective, such events reinforce the importance of a system‐based approach to food safety, in which multiple preventive controls work together to reduce, rather than eliminate, risk. From a consumer perspective, however, these failures may undermine confidence in the institutions responsible for food safety.

So, are consumers truly underestimating food safety when they choose raw milk? Choosing raw milk is not just a food safety issue, it is a window into how consumers perceive modern food systems and all the human factors driving their decision‐making. The persistence of raw milk consumption suggests that traditional risk communication approaches may be insufficient. Posting outbreak numbers, recall notices, and statistics on agency websites alone will not build trust or change decisions shaped by values and heuristics. I suggest that the dairy industry move beyond simply labeling milk as ‘pasteurized’. Clear and accessible communication can alert consumers to risk, but it should be paired with plain‐language explanations of why pasteurization and other food safety‐enhancing treatments are used. I also encourage the industry to better understand why some consumers choose raw milk. Knowing what consumers are looking for can help the industry improve both processing approaches and communication strategies. Trust will not be built by a simple statement that milk is pasteurized. It requires communication that is transparent, scientifically accurate, and close enough to consumers' everyday concerns that the process feels understandable rather than distant or overly complex. Consumers who choose raw milk are not necessarily rejecting the goal of food safety. Instead, they may be using their own reasoning to protect themselves and their families by choosing what they believe is the better food. As food safety professionals, we need to recognize these differences in how people approach food safety and engage with consumers' beliefs, not dismiss them.

## CONFLICT OF INTEREST

There is no conflict of interest.
